# The nature of delayed dream incorporation (‘dream‐lag effect’): Personally significant events persist, but not major daily activities or concerns

**DOI:** 10.1111/jsr.12697

**Published:** 2018-04-22

**Authors:** Jean‐Baptiste Eichenlaub, Elaine van Rijn, Mairéad Phelan, Larnia Ryder, M. Gareth Gaskell, Penelope A. Lewis, Matthew P. Walker, Mark Blagrove

**Affiliations:** ^1^ Swansea University Sleep Laboratory Department of Psychology Swansea University Swansea UK; ^2^ Sleep, Language and Memory Laboratory Department of Psychology University of York York UK; ^3^ School of Psychology and Cardiff University Brain Imaging Centre Cardiff University Cardiff UK; ^4^ Center for Human Sleep Science Department of Psychology University of California Berkeley CA USA

**Keywords:** autobiographical memory, declarative memory, dreaming, memory consolidation, rapid eye movement sleep

## Abstract

Incorporation of details from waking life events into rapid eye movement (REM) sleep dreams has been found to be highest on the 2 nights after, and then 5–7 nights after, the event. These are termed, respectively, the day‐residue and dream‐lag effects. This study is the first to categorize types of waking life experiences and compare their incorporation into dreams across multiple successive nights. Thirty‐eight participants completed a daily diary each evening and a dream diary each morning for 14 days. In the daily diary, three categories of experiences were reported: major daily activities (MDAs), personally significant events (PSEs) and major concerns (MCs). After the 14‐day period each participant identified the correspondence between items in their daily diaries and subsequent dream reports. The day‐residue and dream‐lag effects were found for the incorporation of PSEs into dreams (effect sizes of .33 and .27, respectively), but only for participants (*n *=* *19) who had a below‐median total number of correspondences between daily diary items and dream reports (termed “low‐incorporators” as opposed to “high‐incorporators”). Neither the day‐residue or dream‐lag effects were found for MDAs or MCs. This U‐shaped timescale of incorporation of events from daily life into dreams has been proposed to reflect REM sleep‐dependent memory consolidation, possibly related to emotional memory processing. This study had a larger sample size of dreams than any dream‐lag study hitherto with trained participants. Coupled with previous successful replications, there is thus substantial evidence supporting the dream‐lag effect and further explorations of its mechanism, including its neural underpinnings, are warranted.

## 1 INTRODUCTION

There is evidence for a 7‐day U‐shaped timescale of incorporation of memories of experiences when awake into dreams, in which events from 1 or 2 days before the dream, and from 5 to 7 days before the dream, are preferentially incorporated into dream content (Blagrove, Henley‐Einion, Barnett, Edwards, & Seage, 2011; Blagrove, Fouquet et al., 2011; Nielsen, Kuiken, Alain, Stenstorm, & Powell, 2004; Nielsen & Powell, 1989). The recent incorporations are termed the day‐residue effect, and the delayed incorporations the dream‐lag effect. Nielsen et al. (2004) and Blagrove, Henley‐Einion et al. (2011) have shown that the dream‐lag effect is not a result of confounding of the incorporation of weekly recurring events, where day‐residue incorporations of these might spuriously appear to refer to events from the previous week. The dream‐lag effect has been found using naturalistic events that occur in the participants' daily lives (Blagrove, Fouquet et al., 2011; Blagrove, Henley‐Einion et al., 2011; Nielsen et al., 2004; Nielsen & Powell, 1989, experiment 1; van Rijn et al., 2015, experiment 1, home dreams condition) and using standardized stimulus designs with stimuli such as a night sleeping in the sleep laboratory (Nielsen & Powell, 1989, experiment 2; van Rijn et al., 2015, experiment 2) or the watching of an emotionally arousing videotape of an animal sacrifice (Powell, Nielsen, Cheung, & Cervenka, 1995).

The dream‐lag effect has been found to hold for REM sleep dreams but not N2 dreams (Blagrove, Fouquet et al., 2011) or slow wave sleep (SWS, or N3) dreams (van Rijn et al., 2015). This indicates that the dream‐lag effect might be indexing an approximately 7‐day process of memory consolidation specific to REM sleep (Blagrove, Fouquet et al., 2011), possibly reflecting the gradual transfer of new memory representations from the hippocampus to the neocortex and their integration into older neocortical representations (Nielsen & Stenstrom, 2005). Although non‐REM sleep stages N3 and N2 are believed to play a critical role in memory consolidation (e.g., Diekelmann & Born, 2010; Gais & Born, 2004; Smith, 2001), growing evidence supports the offline benefit of REM sleep for the processing of emotional memories (e.g., Giuditta et al., 1995; Groch, Wilhelm, Diekelmann, & Born, 2013; Nishida, Pearsall, Buckner, & Walker, 2009; Walker & Stickgold, 2010), and the dream‐lag, as an REM sleep phenomenon, might reflect these REM sleep processes.

A process of triage for sleep‐dependent memory consolidation has been proposed (Stickgold & Walker, 2013) in which there is a differential processing of memories based on factors such as salience, emotional involvement and future relevance. The question thus arises of what types of life experience contribute to the dream‐lag effect. Most studies on the dream‐lag effect have allowed for just one overall correspondence score to summarize the comparison of a dream report with a prior waking life event or a diary record of a day's events. The present study instead used a daily diary (from Fosse, Fosse, Hobson, & Stickgold, 2003) that differentiates between major daily activities (MDAs), personally significant events (PSEs) and major concerns (MCs), so as to allow for the identification of incorporation of three different types of waking life event into dream reports. Participants were thus allowed to score multiple correspondences between each daily diary and each dream report. Using this approach, van Rijn et al. (2015, experiment 1) reported the dream‐lag effect for PSEs, but not for MDAs or MCs, for REM dream reports that were elicited from instrumental awakenings across one night at home.

Importantly, Henley‐Einion and Blagrove (2014), using such a multiple‐correspondences method, highlighted individual differences in overall number of correspondences identified by each participant between their diary records and dream reports. This individual difference in tendency to find connections between daily life and dream reports was found to result in a dilution or eradication of time‐course relationships for individuals who identify high numbers of such incorporations. The authors thus recommended dividing participants in such multiple‐correspondences studies into two groups, using a median split based on the total number of correspondences identified by each participant across the whole study. This approach was subsequently supported by van Rijn et al.'s (2015) study, where a significant (U‐shaped) difference in number of incorporations was found for PSEs, but only for the participants who identified a below‐median total number of correspondences. Accordingly, low and high incorporators were analysed separately in the present study and we hypothesized that there would be a dream‐lag effect in the low‐incorporator subsample only, and only for PSEs.

Although many studies have demonstrated the dream‐lag effect using naturalistic diary keeping, this has been despite the variability in types and salience of events across the days during which the diary was kept. The effect of this variability can be reduced by having a large number of daily diaries and dream reports, collected over many days, resulting in a large matrix of dream report and daily diary comparisons, as in Blagrove, Henley‐Einion et al. (2011). The current study was the first to couple the multiple‐correspondences method from van Rijn et al. (2015) with the matrix comparison method as described in Blagrove, Henley‐Einion et al. (2011).

The main hypothesis was tested using the participants' scores for number of incorporations of waking life experiences in dream reports, as a function of number of days between the diary entry and the occurrence of the dream. Most studies on the dream‐lag have similarly used participants' scores, given the personal knowledge sometimes needed to identify incorporation of waking life experiences, but some have used independent judges' scores. Where a standardized experimental stimulus was presented, scores collected from judges have evidenced the dream‐lag effect (Nielsen & Powell, 1989, experiment 2; Powell et al., 1995; van Rijn et al., 2015, experiment 2). However, when experiments have been naturalistic, using daily diaries to record waking life experiences, independent judges have not identified the dream‐lag effect. In Nielsen and Powell (1992), two independent judges failed to evidence the dream‐lag effect, with the inter‐judge agreement and mean number of incorporations they identified being low. Furthermore, in Blagrove, Henley‐Einion et al. (2011), the dream‐lag effect was only evidenced when participants assessed their own incorporations and not when independent judges assessed them. Problems with the use of independent judges for scoring dream reports in general are detailed more extensively in Sikka, Valli, Virta, and Revonsuo (2014). As a result of these findings we did not employ independent judges in the current study.

In summary, the present study tested the hypothesis that the dream‐lag effect will occur for personally significant events (PSEs), but only for participants with a below‐median total number of incorporations. This study aimed to compile the largest sample of dream reports versus daily diary combinations yet attempted in a study of the dream‐lag, where participants are also given training and criteria to identify individual correspondences between dream reports and daily diaries. This large matrix of dream report and daily diary combinations was used so as to enhance the robustness of the results and to reduce potential confounding as a result of participants possibly having some diary days of particularly high salience.

## 2 METHOD

### 2.1 Participants

Forty‐four healthy volunteers (seven male, 37 female; aged 17–64 years, mean age = 24.1, *SD* = 9.6) were recruited to the study. Participants were those who self‐reported frequently recalling dreams (defined as recalling dreams 5–7 days per week), sleeping a minimum of 7 hr per night (with no disorders that could affect their sleep), not taking recreational drugs and not having an excessive alcohol intake (defined as intake greater than 6 units of alcohol per night or greater than 21 units per week). Participants gave written informed consent to take part in the study and were paid for their participation. Ethical approval for the study was obtained from the Research Ethics Committee of the School of Human and Health Sciences, Swansea University.

Of the 44 participants, one participant did not complete the diaries properly (skipped several daily diaries and dream reports) and five participants left the experiment before the end. Thirty‐eight participants (five male, 33 female; mean age = 23.9 years, *SD* = 9.4) were thus considered for further analysis.

### 2.2 Procedure and materials

#### 2.2.1 Daily diaries

Participants were instructed to keep a daily diary for 14 consecutive days. The daily diary was taken from Fosse et al. (2003). Each evening participants recorded information in the diary about their experiences during the day for the following three categories.

1. Major daily activities (MDAs): activities that took up most of the participants' time during the day (e.g. going to work or university, meals and shopping).
2. Personally significant events (PSEs): important daily events that may or may not have taken up much time (e.g. emotional events).
3. Major concerns (MCs): concerns or thoughts that participants had on their mind during the day that may not have taken up much time but were still considered important to them (e.g. money problems or exam stress).

Up to five items could be recorded in each category. For each item reported, participants were also instructed to state any accompanying emotion (e.g. anger, anxiety/fear, sadness, shame, joy/elation, love/erotic, and surprise) and to rate the intensity of the emotion on a scale from 1 (low) to 3 (high).

#### 2.2.2 Dream reports

Participants were instructed to keep a home dream diary during the same 14‐day period (the first dream report being from the night that followed the first daily diary). Each morning they were instructed to type out a report on any dreams they had during the night. Participants were asked to describe these in as much detail as they could remember, including a description of the setting, characters, objects and feelings. As with the daily diary, they were also instructed to report any accompanying emotion and to rate the intensity of the emotion on a scale from 1 (low) to 3 (high). If they had more than one dream in a night, they were asked to describe each dream separately. A digital voice recorder was given to assist in completing the dream diary. At the end of the 14 days participants returned their daily diaries and dream diaries by Email to the investigators.

### 2.3 Correspondence identification task

**Figure 1:**
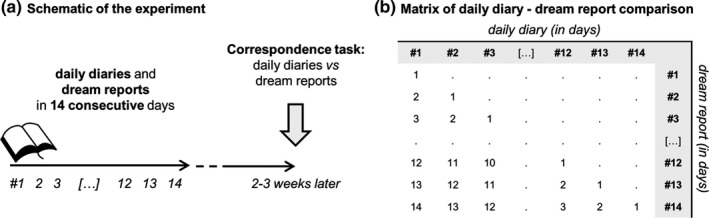
(a) Schematic of the experiment. Participants kept a daily diary each evening and a dream diary every morning for 14 consecutive days. Approximately 2–3 weeks later, participants identified correspondences between the daily diary items and the subsequent dream reports. (b) Matrix of daily diary and dream report comparisons. For each dream report, the daily diaries from the preceding days were considered. The 1 day period refers to the daily diaries being completed on the day up to the night of the dream (e.g., daily diary #3 and dream report #3; daily diary #12 and dream report #12), the 2 days period refers to the daily diaries being completed on the day before this, that is, 2 days before the night or morning of the dream (e.g., daily diary #1 and dream report #2; daily diary #10 and dream report #11), and so on

Approximately 2–3 weeks after the end of the 14‐day period, participants were sent materials so that they could perform the correspondence identification task (see Figure 1a). Participants were instructed to compare each of their 14 daily diary entries with each of their subsequent dream report transcripts in order to identify similarities or correspondences between the diary items and dream reports, such as the characters, objects, actions, locations or themes. For this task, participants were presented with a randomized series of A3 sheets (42.0 × 29.7 cm), with a daily diary on the left side and a dream report on the right side of each sheet. The number of sheets completed ranged from 60 to 105 sheets (mean = 89.7, *SD* = 14.2), depending upon each participant's number of dream reports.

If participants identified a correspondence, they were instructed to draw boxes around the matched words or sentences in the daily diary and around the matched words or sentences in the dream report, and then to rate the level of correspondence between the two parts using a scale from 0 (none) to 8 (extremely strong). Zero, one or more than one correspondence could be identified for each A3 sheet.

#### 2.3.1 Correspondence task training instructions

All participants received training instructions on how to identify correspondences between dream contents and daily diary items. Instructions stated that these can be: (i) literal correspondences, where the same characteristic occurs in the diary item as in the dream report; (ii) weak correspondences, where there is some similarity between a diary item and the dream report; (iii) personal correspondences, where personal background knowledge provides a link between the diary item and the dream report, and (iv) symbolic correspondences, where the link between the dream report and the diary item is abstract or metaphorical. Participants then answered the following question for each correspondence they identified between a diary item and dream report: “What is the extent of correspondence between the part of your daily log and the part of the dream report that you have put in boxes?” They scored each correspondence on a 9‐point scale, from 0 to 8, which had anchor points: 0 = none; 2 = weak; 4 = moderate; 6 = strong; 8 = extremely strong.

### 2.4 Data analysis

The length of each dream report in words was assessed following Antrobus' (1983) definition: “the count of all words in sentences or phrases in which the subject was describing something that had occurred just before waking. It excluded ‘ahs', ‘uhms’, repeated and corrected words, and all commentary on the experience, the report, or the current status of the subject”. Only dream reports of 10 words or more were included in the analysis, as short dream reports might indicate a failure of optimal recording.

For each sheet showing a daily diary and dream report combination, the total number of incorporations identified by the participant was summed for each of the three diary categories. For each participant, the mean number of incorporations for each of the three diary categories was then computed for each day period, defined as the number of days between the diary day and the occurrence of the dream. For this, the 1‐day period refers to the diary being completed on the day before the night of the dream (e.g. daily diary #3 and dream report #3; daily diary #12 and dream report #12), the 2‐day period refers to the daily diary being completed on the day before this, that is, 2 days before the night or morning of the dream (e.g. daily diary #1 and dream report #2; daily diary #10 and dream report #11), and so on (see Figure 1b). This method of analysis produces a timescale of mean number of incorporations per sheet (effectively, per dream) as a function of the number of days between the diary day and the occurrence of the dream. That is, the method shows how many incorporations occur in dreams from each of the preceding diary days.

Using this approach, the 1‐day period has the largest number of correspondence sheets; that is, daily diary #1 and dream report #1, daily diary #2 and dream report #2, …, daily diary #14 and dream report #14 (i.e. there are 14 correspondence sheets in total for the 1‐day period). Importantly, as the period between the daily diary entry and dream occurrence increases, the number of sheets from which data can be derived decreases, and so, for the 14‐day period, there is only one correspondence sheet (i.e. daily diary #1 and dream report #14). Accordingly, and so as to ensure a sample of at least several correspondence sheets for each time period in the analysis, only periods up to 12 days between the diary day and dream occurrence were analysed.

Across participants, the mean number of incorporations for each day‐period (i.e. 1 day, 2 days, …., 11 days, 12 days, between diary entry and dream occurrence) and for each daily diary category was calculated. For inferential statistics, a Friedman test was conducted to assess whether the 12 day‐means (i.e., 1 day, 2 days, …., 11 days, 12 days) differ from each other. A non‐parametric test was used as almost half the variables (47.2%) were non‐normally distributed on a Shapiro‐Wilk test, and the majority of day variables for PSEs were not normally distributed. Where the Friedman test was significant, a Wilcoxon signed‐rank test was used to compare means of the three combined time periods that are used to test for the day‐residue and dream‐lag effects, namely the mean of 1 and 2 days, mean of 3 and 4 days, and mean of 5, 6 and 7 days. These periods were defined in advance and in accordance with the literature (Blagrove, Fouquet et al., 2011; Nielsen et al., 2004; van Rijn et al., 2015, 2018). Day‐residue and dream‐lag effects were predicted for the incorporation of PSEs; hence, the means of the number of PSE incorporations for the periods 1–2 days and 5–7 days were each hypothesized to be greater than for the period 3–4 days. Effect sizes were calculated as *z*/√(*n*) where *z* is the *z*‐statistic and *n* the number of observations (i.e. 38 per subgroup). These effects were hypothesized to occur for participants with a below‐median total number of incorporations. To split the participants into low and high incorporators, the mean number of all correspondences per dream across the whole study was calculated for each of the 38 participants; a median split was then performed.

## 3 RESULTS

**Figure 2:**
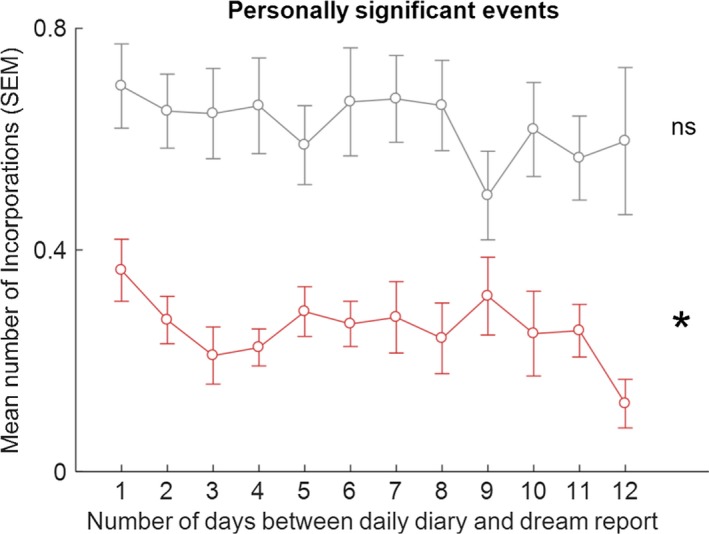
Means (SEM) of number of incorporations of PSEs into dreams, for the low‐ (in red) and high‐ (in grey) incorporators, as a function of number of days between daily diary and dream report. *p = .025, (Friedman test); ns, non‐significant

**Figure 3:**
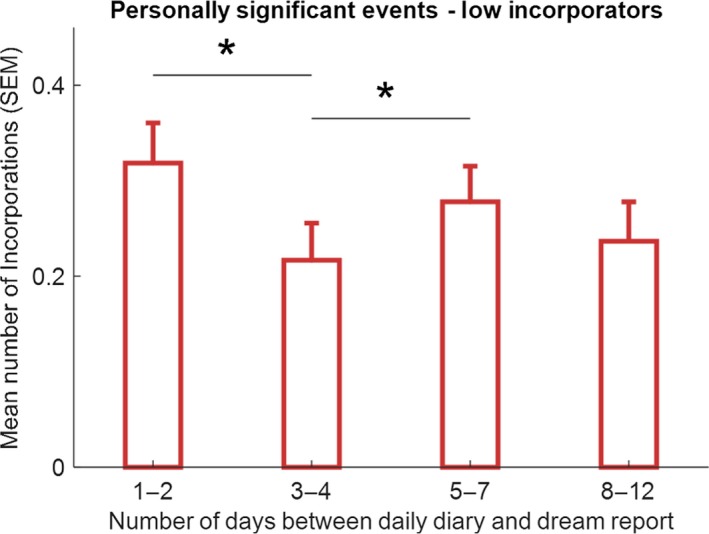
Means (SEM) of number of incorporations of PSEs into dreams for combined timescale periods for low‐incorporators. *p < .05, (Wilcoxon test, one‐tailed)

**Figure 4:**
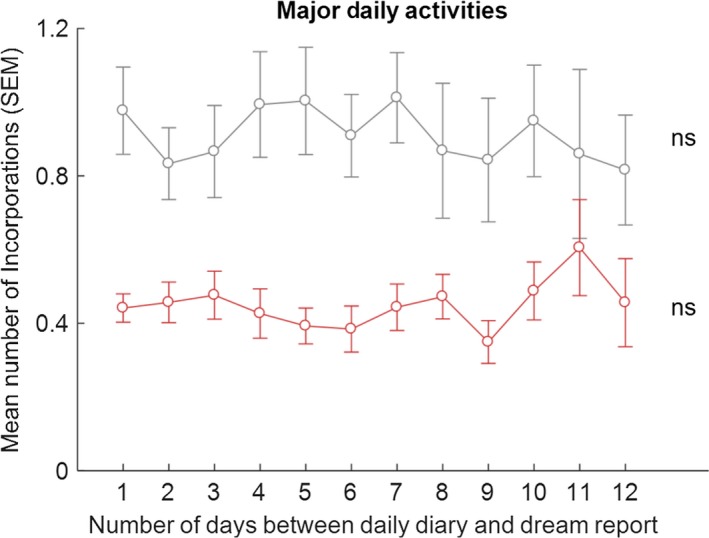
Means (SEM) of number of incorporations of MDAs into dreams, for the low‐ (in red) and high‐ (in grey) incorporators, as a function of number of days between daily diary and dream report; ns, non‐significant (Friedman test)

**Figure 5:**
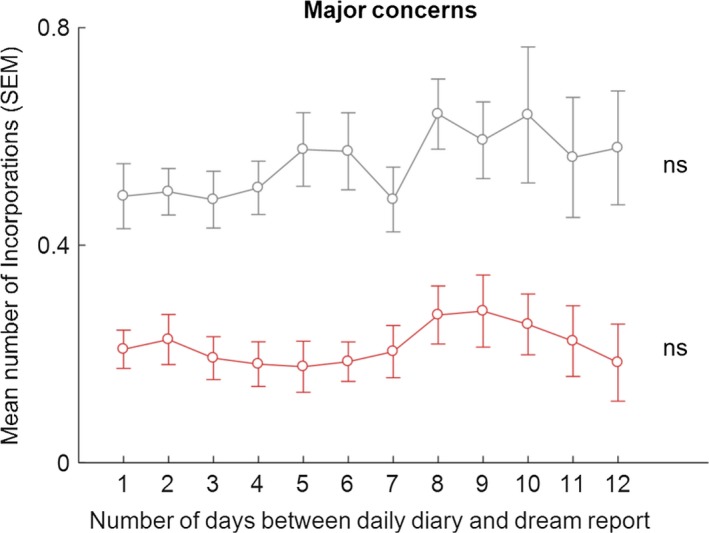
Means (SEM) of number of incorporations of MCs into dreams, for the low‐ (in red) and high‐ (in grey) incorporators, as a function of number of days between daily diary and dream report; ns, non‐significant (Friedman test)

Participants recorded on average 4.07 (*SD* = 0.77) MDAs, 2.69 (1.40) PSEs and 2.41 (1.42) MCs, per diary day. The mean and median numbers of all correspondences per dream were calculated for the 38 participants (mean = 1.31, *SD* = 0.67, minimum = 0.24, maximum = 3.62, median = 1.16). The median split resulted in 19 low (below median) and 19 high (above median) incorporators. Summary data for the total number of incorporations per dream for these groups were: low incorporators, mean = 0.80, *SD* = 0.27, minimum = 0.24, maximum = 1.14; high incorporators, mean = 1.82, *SD* = 0.55, minimum = 1.18, maximum = 3.62). The number of incorporations of PSEs, MDAs and MCs, as a function of time between the diary entry and dream occurrence, for high and low incorporators separately, are shown in Figures 2, 3, 4, 5.

### 3.1 Personally Significant Events (PSEs)

As hypothesized, low incorporators exhibited a significant difference between the mean number of incorporations per day for PSEs across the 12 days (Friedman test, χ^2^(11) = 21.905, *p* = .025; Figure 2), which followed the U‐shaped timescale found in the literature. In contrast, high incorporators did not exhibit a significant difference between the mean number of incorporations per day for PSEs across the 12 days (Friedman test, χ^2^(19) = 11.817, *p* = .378).

In order to compare means for PSEs across the timescale in low incorporators, means for the standard combined periods were computed (1–2 days; 3–4 days; 5–7 days; day 8 and above, here 8–12 days) and are shown in Figure 3. As the variables were not normally distributed (high correspondences, all Shapiro‐Wilk statistics >.94, all *p* >.270, *df* = 19 for all tests; low correspondences, all Shapiro‐Wilk statistics >.90, all *p* >.074, *df* = 19 for all tests), non‐parametric tests were used for comparisons. The mean number of incorporations per day for the combined period 5–7 days was significantly higher than for the period 3–4 days (Wilcoxon test, *z* = 1.690, *p* = .046, one‐tailed, effect size = .27), demonstrating a dream‐lag effect. In addition, the mean number of incorporations per day for the period 1–2 days was significantly higher than for the period 3–4 days (Wilcoxon test, *z* = 2.012, *p* = .022, one‐tailed, effect size = .33), demonstrating a day‐residue effect. All other comparisons were non‐significant.

As the 8–12 days mean was not significantly different from both the 3–4 days and 5–7 days means for the low‐incorporator group, we conducted exploratory analyses on each individual day period from the 8‐day to the 12‐day period to test whether the dream‐lag effect was observed beyond the 5–7‐day period. All comparisons of the 3–4‐day period with each individual day period from 8 to 11 were not significant (Wilcoxon tests, all *z* < 1.066, all *p *> .286, two‐tailed), as were all comparisons of the 5–7‐day period with each individual day period from 8 to 11 (Wilcoxon tests, all *z *< 0.967, all *p* > .333, two‐tailed). However, the comparison of 5–7 days with the 12‐day period was significant (Wilcoxon test, *z* = 2.938, *p* = .003, two‐tailed), whereas the comparison of the 3–4 day period with the 12‐day period was not (Wilcoxon test, *z* = 1.728, *p* = .084, two‐tailed).

The dream‐lag effect was shown for the low incorporators but not the high incorporators by non‐parametric tests applied separately to the two groups. So as to provide a direct statistical comparison between the groups, the 5–7‐day PSE incorporation scores for the groups, standardized by subtracting their number of 3–4‐day PSE incorporations, were compared. The low incorporators had significantly higher standardized 5–7‐day incorporations than did the high incorporators (means = 0.061 [*SD* = 0.140], and −0.010 [0.203], respectively, Mann–Whitney *U* = 124.00, z = 1.650, *p* = .049, one‐tailed). The low incorporators also had higher standardized day‐residue (1–2 days) incorporations than did the high incorporators (means = 0.102 [*SD* = 0.201] and 0.020 [0.230], respectively), but this difference was not significant (Mann–Whitney *U* = 145.00, z = 1.036, *p* = .150, one‐tailed).

### 3.2 Major Daily Activities (MDAs)

As hypothesized, there were no significant differences in incorporation of MDAs for low (Friedman test, χ^2^(11) = 7.084, *p* = .792) and high incorporators (Friedman test, χ^2^(11) = 13.344, *p* = .271; Figure 4).

### 3.3 Major Concerns (MCs)

Similarly, there were no significant differences in incorporation of MCs for low (Friedman test, χ^2^(11) = 9.340, *p* = .591) and high incorporators (Friedman test, χ^2^(11) = 9.781, *p* = .550; Figure 5).

## 4 DISCUSSION

As hypothesized, the dream‐lag effect was found for personally significant experiences (PSEs) for the below‐median correspondences group (i.e. low incorporators), whereas neither major daily activities (MDAs) nor major concerns (MCs) evidenced the day‐residue or dream‐lag effects. The lack of day‐residue and dream‐lag effects for MDAs and MCs may be because these types of life experiences are not temporally exact or distinctive enough to show these effects, in contrast to PSEs that are salient and more easily localizable in time. Alternatively, if delayed incorporation into dreams reflects REM sleep‐dependent memory consolidation, the absence of a dream‐lag for MDAs and MCs suggests that, contrary to personally significant events, major activities and concerns might not be subject to REM sleep‐dependent memory consolidation.

The review at the start of the introduction listed eight studies that evidence the dream‐lag effect. However, some other studies have failed to replicate the effect. These studies are as follows, with possible reasons for non‐replication: Nielsen and Powell (1992), where judges were used in a naturalistic study and hence might not have had sufficient personal knowledge of the dreamer to do the task; van Rijn et al.'s (2015) experiment 1, laboratory awakenings group, where SWS had been disrupted by instrumental awakenings prior to the REM awakenings; Henley‐Einion and Blagrove (2014), where the diary method and analysis conflated all daily experiences and so did not distinguish between PSEs, MDAs and MCs; van Rijn et al. (2018), where there were only one or two REM dreams per participant; and Schredl (2006), where no criteria were given to participants as to how to determine the correspondences between dream content and daytime events. In contrast, the current study confirms the dream‐lag in a design that uses the largest number of dream reports (212, for the below median correspondences group), and largest number of daily diary and dream report combinations, for any dream‐lag study hitherto where criteria were given for comparing dream reports with waking life experiences. We acknowledge a shortcoming of the current study is that dream reports were collected from spontaneous home awakenings and so the sleep stage at awakening is not known. However, because REM sleep prevails at the end of the night it was expected that in most instances recall in the morning would be from REM sleep.

Of note is that whereas the onset of the 5–7‐day dream‐lag was easily outlined, after this it was only at the 12‐day period that incorporations were significantly less than for the 5–7‐day period. The previous literature is unclear on levels of incorporation after the 5–7‐day period. Whereas Blagrove, Fouquet et al. (2011) reported a significant decline in the number of incorporations between the 5–7‐day and 8–9‐day periods, Blagrove, Henley‐Einion et al. (2011) and van Rijn et al. (2015) did not report a significant difference between incorporations in the 5–7‐day period and in the 8–12‐day and 8–9‐day periods, respectively. However, in the current study each of the 8–12‐day periods analysed separately did not have significantly greater levels of incorporation than the 3–4‐day comparison period, which accords with the previous experimental literature, thus supporting the definition of the dream‐lag period as 5–7 days after events.

In the current study and in van Rijn et al. (2015) the dream‐lag was only found for individuals low in total number of correspondences, and thus sparing in their identification of correspondences. Henley‐Einion and Blagrove (2014) proposed that high incorporators have, for undetermined personality and cognitive reasons, such as over‐inclusion or even confabulation, a tendency to identify a large number of correspondences, such as occurs in the Barnum effect. The correspondence task used here, by allowing multiple matches between the daily diary and the dream report, contrasts with earlier dream‐lag studies where participants were asked for only one score to give a single measure of the similarity between a dream report and a daily life report. For high incorporators the present multiple matches design leads to a dilution or eradication of the dream‐lag effect, and even of the highly robust day‐residue effect. Further studies are needed to explore this inter‐subject variability in the tendency to find connections between daily life and dream reports, including reference to individual differences in affirmative bias (Blagrove, French, & Jones, 2006).

Given the findings in the current study and the previous successful replications, the dream‐lag effect does seem to be well evidenced, and the neural and physiological mechanisms of the dream‐lag effect now need to be determined. Future research should address whether the dream‐lag indexes memory reactivation, lability and reconsolidation, which have been hypothesized to enable updating or mismatch identification during consolidation (Wang & Morris, 2011), and which may hence indicate a behavioural or brain function that could result in delayed incorporations into dreams. One possibility is that the dream‐lag reflects (i.e. is the mental experience of) the endogenous reactivation of memory during sleep, which has emotional memory or integrative learning functions, as proposed by Oudiette and Paller (2013). Support for the relationship of dream content to memory consolidation occurring during sleep is provided by Perogamvros and Schwartz (2012), Wamsley (2014), Wamsley, Tucker, Payne, Benavides, and Stickgold (2010) and Wamsley, Perry, Djonlagic, Babkes Reaven, and Stickgold (2010). As argued by Wamsley and Stickgold (2011), dreaming may be the experience of memory consolidation (during sleep see also Blagrove, Ruby, & Eichenlaub, 2013; Eichenlaub, Cash, & Blagrove, 2017; for critical discussions). Whereas it is arguable that day‐residues reflect waking life experiences with no functional purpose (De Koninck, Wong, & Hébert, 2012), it seems difficult to devise a non‐functional process that can result in these delayed incorporations, and Nielsen and Stenstrom (2005) proposed a physiological reason for the 5–7‐day delayed incorporation, involving the gradual transfer of new memories from the hippocampus to the neocortex over a period of about one week.

To summarise, this study is the first to combine the strengths of the designs used in van Rijn et al. (2015) and Blagrove, Henley‐Einion et al. (2011), so that a large number of multiple comparisons between daily diary categories and a series of dreams are conducted. The results here, coupled with previous successful replications, provide substantial evidence in favour of the dream‐lag effect and of it being specific to personally significant events, and indicate that further explorations of its mechanisms are warranted.

## CONFLICT OF INTEREST

No conflicts of interest declared.

## AUTHOR CONTRIBUTIONS

Study design: JBE, MGG, PAL, MPW and MB. Data acquisition: JBE, EvR, MP and LR. Data analysis: JBE, EvR, MP, LR and MB. Interpretation of data: JBE, EvR, MGG, PAL, MPW and MB. Manuscript preparation: JBE, EvR, MGG, PAL, MPW and MB.

## References

[jsr12697-bib-0001] Antrobus, J. (1983). REM and NREM sleep reports: Comparison of word frequencies by cognitive class. Psychophysiology, 20, 562–568. 10.1111/j.1469-8986.1983.tb03015.x 6635096

[jsr12697-bib-0002] Blagrove, M. , Fouquet, N. C. , Henley‐Einion, J. A. , Pace‐Schott, E. F. , Davies, A. C. , Neuschaffer, J. L. , & Turnbull, O. H. (2011). Assessing the dream‐lag effect for REM and NREM stage 2 dreams. PLoS ONE, 6, e26708 10.1371/journal.pone.0026708 22046336 PMC3202556

[jsr12697-bib-0003] Blagrove, M. , French, C. C. , & Jones, G. (2006). Probabilistic reasoning, affirmative bias and belief in precognitive dreams. Applied Cognitive Psychology, 20, 65–83. 10.1002/acp.1165

[jsr12697-bib-0004] Blagrove, M. , Henley‐Einion, J. , Barnett, A. , Edwards, D. , & Seage, C. H. (2011). A replication of the 5–7 day dream‐lag effect with comparison of dreams to future events as control for baseline matching. Consciousness and Cognition, 20, 384–391. 10.1016/j.concog.2010.07.006 20739193

[jsr12697-bib-0005] Blagrove, M. , Ruby, P. , & Eichenlaub, J.‐B. (2013). Dreams are made of memories, but maybe not for memory. Behavioral and Brain Sciences, 36, 609–610. 10.1017/S0140525X13001222 24304749

[jsr12697-bib-0006] De Koninck, J. , Wong, C. , & Hébert, G. (2012). Types of dream incorporations of language learning and learning efficiency. Journal of Sleep Research, 21(Suppl. S1): 190 10.1111/j.1365-2869.2012.01044.x

[jsr12697-bib-0007] Diekelmann, S. , & Born, J. (2010). The memory function of sleep. Nature Reviews Neuroscience, 11, 114–126. 10.1038/nrn2762 20046194

[jsr12697-bib-0008] Eichenlaub, J.‐B , Cash, S. S. , & Blagrove, M. (2017) Daily life experiences in dreams and sleep‐dependent memory consolidation In: Axmacher N. & Rasch B. (Eds.) Cognitive neuroscience of memory consolidation (pp. 161–172). Switzerland: Springer Cham 10.1007/978-3-319-45066-7_10

[jsr12697-bib-0009] Fosse, M. J. , Fosse, R. , Hobson, J. A. , & Stickgold, R. J. (2003). Dreaming and episodic memory: A functional dissociation? Journal of Cognitive Neuroscience, 15, 1–9. 10.1162/089892903321107774 12590838

[jsr12697-bib-0010] Gais, S. , & Born, J. (2004). Multiple processes strengthen memory during sleep. Psychologica Belgica, 44, 105–120. 10.1101/lm.77104

[jsr12697-bib-0011] Giuditta, A. , Ambrosini, M. V. , Montagnese, P. , Mandile, P. , Cotugno, M. , Zucconi, G. G. , & Vescia, S. (1995). The sequential hypothesis of the function of sleep. Behavioral Brain Research, 69, 157–166. 10.1016/0166-4328(95)00012-I 7546307

[jsr12697-bib-0012] Groch, S. , Wilhelm, I. , Diekelmann, S. , & Born, J. (2013). The role of REM sleep in the processing of emotional memories: Evidence from behavior and event‐related potentials. Neurobiology of Learning and Memory, 99, 1–9. 10.1016/j.nlm.2012.10.006 23123802

[jsr12697-bib-0013] Henley‐Einion, J. , & Blagrove, M. (2014). Assessing the day residue and dream‐lag effects using the identification of multiple correspondences between dream reports and waking life diaries. Dreaming, 24, 71–88. 10.1037/a0036329

[jsr12697-bib-0014] Nielsen, T. A. , Kuiken, D. , Alain, G. , Stenstorm, P. , & Powell, R. A. (2004). Immediate and delayed incorporations of events into dreams: Further replication and implications for dream function. Journal of Sleep Research, 13, 327–336. 10.1111/j.1365-2869.2004.00421.x 15560767

[jsr12697-bib-0015] Nielsen, T. A. , & Powell, R. A. (1989). The ‘dream‐lag’ effect: A 6‐day temporal delay in dream content incorporation. Psychiatric Journal of the University of Ottawa, 14, 562–565. 2813638

[jsr12697-bib-0016] Nielsen, T. A. , & Powell, R. A. (1992). The day‐residue and dream‐lag effects: A literature review and limited replication of two temporal effects in dream formation. Dreaming, 2, 67–77. 10.1037/h0094348

[jsr12697-bib-0017] Nielsen, T. A. , & Stenstrom, P. (2005). What are the memory sources of dreaming? Nature, 437(7063), 1286–1289. 10.1038/nature04288 16251954

[jsr12697-bib-0018] Nishida, M. , Pearsall, J. , Buckner, R. L. , & Walker, M. P. (2009). REM sleep, prefrontal theta, and the consolidation of human emotional memory. Cerebral Cortex, 19, 1158–1166. 10.1093/cercor/bhn155 18832332 PMC2665156

[jsr12697-bib-0019] Oudiette, D. , & Paller, K. A. (2013). Upgrading the sleeping brain with targeted memory reactivation. Trends in Cognitive Sciences, 17, 142–149. 10.1016/j.tics.2013.01.006 23433937

[jsr12697-bib-0020] Perogamvros, L. , & Schwartz, S. (2012). The roles of the reward system in sleep and dreaming. Neuroscience and Biobehavioral Reviews, 36, 1934–1951. https://doi.org/S0149-7634(12)00089-9 22669078 10.1016/j.neubiorev.2012.05.010

[jsr12697-bib-0021] Powell, R. A. , Nielsen, T. A. , Cheung, J. S. , & Cervenka, T. M. (1995). Temporal delays in incorporation of events into dreams. Perceptual and Motor Skills, 81, 95–104. 10.2466/pms.1995.81.1.95 8532489

[jsr12697-bib-0022] van Rijn, E. , Eichenlaub, J. , Lewis, P. , Walker, M. , Gaskell, M. , Malinowski, J. , & Blagrove, M. (2015). The dream‐lag effect: Selective processing of personally significant events during Rapid Eye Movement sleep, but not during Slow Wave Sleep. Neurobiology of Learning and Memory, 122, 98–109. 10.1016/j.nlm.2015.01.009 25683202

[jsr12697-bib-0023] van Rijn, E. , Reid, A. M. , Edwards, C. L. , Malinowski, J. E. , Ruby, P. M. , Eichenlaub, J.‐B. , & Blagrove, M. T. (2018). Daydreams incorporate recent waking life concerns but do not show delayed (‘dream‐lag’) incorporations. Consciousness and Cognition, 58, 51–59. 10.1016/j.concog.2017.10.011 29128282

[jsr12697-bib-0024] Schredl, M. (2006). Factors affecting the continuity between waking and dreaming: Emotional intensity and emotional tone of the waking‐life event. Sleep Hypnosis, 8, 1–5.

[jsr12697-bib-0025] Sikka, P. , Valli, K. , Virta, T. , & Revonsuo, A. (2014). I know how you felt last night, or do I? Self‐ and external ratings of emotions in REM sleep dreams. Consciousness and Cognition, 25, 51–66. 10.1016/j.concog.2014.01.011 24565868

[jsr12697-bib-0026] Smith, C. (2001). Sleep states and memory processes in humans: Procedural versus declarative memory systems. Sleep Medicine Reviews, 5, 491–506. 10.1053/smrv.2001.0164 12531156

[jsr12697-bib-0027] Stickgold, R. , & Walker, M. P. (2013). Sleep‐dependent memory triage: Evolving generalization through selective processing. Nature Neuroscience, 2013(16), 139–145. 10.1038/nn.3303 PMC5826623 23354387

[jsr12697-bib-0028] Walker, M. P. , & Stickgold, R. (2010). Overnight alchemy: Sleep‐dependent memory evolution. Nature Reviews Neuroscience, 11, 218 10.1038/nrn2762-c1 PMC2891532 20168316

[jsr12697-bib-0029] Wamsley, E. J. (2014). Dreaming and offline memory consolidation. Current Neurology and Neuroscience Reports, 14, 433 10.1007/s11910-013-0433-5 24477388 PMC4704085

[jsr12697-bib-0030] Wamsley, E. J. , Perry, K. , Djonlagic, I. , Babkes Reaven, L. , & Stickgold, R. (2010). Cognitive replay of visuomotor learning at sleep onset: Temporal dynamics and relationship to task performance. Sleep, 33, 59–68. 20120621 10.1093/sleep/33.1.59 PMC2802248

[jsr12697-bib-0031] Wamsley, E. J. , & Stickgold, R. (2011). Memory, sleep and dreaming: Experiencing consolidation. Sleep Medicine Clinics, 6, 97–108. 21516215 10.1016/j.jsmc.2010.12.008 PMC3079906

[jsr12697-bib-0032] Wamsley, E. J. , Tucker, M. , Payne, J. D. , Benavides, J. A. , & Stickgold, R. (2010). Dreaming of a learning task is associated with enhanced sleep‐dependent memory consolidation. Current Biology, 20, 850–855. 10.1016/j.cub.2010.03.027 20417102 PMC2869395

[jsr12697-bib-0033] Wang, S. H. , & Morris, R. G. M. (2011). Hippocampal‐neocortical interactions in memory formation, consolidation, and reconsolidation. Annual Review of Psychology, 61, 49–79. 10.1146/annurev.psych.093008.100523 19575620

